# Modeling *E. coli* Tumbles by Rotational Diffusion. Implications for Chemotaxis

**DOI:** 10.1371/journal.pone.0035412

**Published:** 2012-04-18

**Authors:** Jonathan Saragosti, Pascal Silberzan, Axel Buguin

**Affiliations:** Laboratoire Physico-chimie Curie - UMR 168, Institut Curie, Centre de Recherche – CNRS – UPMC, Paris, France; University of Manchester, United Kingdom

## Abstract

The bacterium *Escherichia coli* in suspension in a liquid medium swims by a succession of runs and tumbles, effectively describing a random walk. The tumbles randomize incompletely, *i.e.* with a directional persistence, the orientation taken by the bacterium. Here, we model these tumbles by an active rotational diffusion process characterized by a diffusion coefficient and a diffusion time. In homogeneous media, this description accounts well for the experimental reorientations. In shallow gradients of nutrients, tumbles are still described by a unique rotational diffusion coefficient. Together with an increase in the run length, these tumbles significantly contribute to the net chemotactic drift via a modulation of their duration as a function of the direction of the preceding run. Finally, we discuss the limits of this model in propagating concentration waves characterized by steep gradients. In that case, the effective rotational diffusion coefficient itself varies with the direction of the preceding run. We propose that this effect is related to the number of flagella involved in the reorientation process.

## Introduction

Micro-organisms living in liquids have developed several strategies to explore their environment and colonize new niches more favorable (nutrients, temperature, oxygen…) for their development [1], [2], [3]. One of the most studied organisms, in this respect, is probably the petrichously flagellated bacterium *Escherichia coli* whose motion is classically described by a succession of runs and tumbles [4]. When all the flagellar motors rotate counterclockwise (CCW), the flagella form a bundle that propels the bacterium in a straight line (“runs”) at constant velocity (*V_run_∼20 µm/s* [5], [6]). When one or several of these motors change direction, which is stochastically determined, flagella unravel from the bundle and the bacterium rotates without advancing (“tumbles”). Run and tumble durations are exponentially distributed with mean durations that are respectively *<τ_run_>∼1 s* and *<τ_tumble_>∼0.1 s* [6]. Furthermore, it has been noticed in [7] that the reorientations during tumbles were not fully random and that it was necessary to introduce a directional persistence (or a memory in the orientation between two successive runs) to describe the 3D trajectory of the bacterium. In any case, the trajectory can be described by a persistent random walk characterized by an active translational diffusion coefficient *D_t_∼V_run_^2^<τ_run_>∼100 µm^2^/s* [8], [9] comparable to the one of a small molecule experiencing Brownian motion [10].

When swimming in a gradient of chemoattractant, *E. coli* performs a temporal comparison of the molecular concentration via its membrane-bound chemoreceptors (tar, tsr, tap,…) [11] that act on the Che pathway [12] to modulate the switching frequency of the fagellar motors, which in turn sets the tumbling frequency [13]. As a consequence, runs are in average longer in the favorable direction and the resulting biased random walk drives the bacterium up the gradient. In this classical description, the tumbles do not actively participate to the chemotaxis phenomenon.

However, since runs and tumbles modulations involve the same pathway, it is natural to think that an increase in run durations could go with a variation (a decrease for synergy) of tumble durations (less reorientation meaning longer persistence) leading to a more efficient motion of the bacteria toward favorable niches. This idea has been supported by numerical simulations [14] and recently confirmed by an experimental study of bacterial trajectories [15].

In the present article, we investigate in details the physical mechanism of tumbling and its apparent resemblance with a process of active rotational diffusion. We first test this idea on bacteria swimming in a homogeneous medium and on tumbler mutants. This description having potentially profound consequences on the chemotaxis of these organisms, we have checked whether a constant diffusion coefficient can account for the differential modulation of the reorientations observed in static shallow nutrient gradients. We end by discussing the limits of this approach in the case of propagating concentration waves where gradients are steeper and imposed locally by the bacteria themselves.

## Results

### Rotational diffusion of a Brownian particle

The motion of ellipsoidal brownian particles dispersed in a liquid medium at rest consists in both translational and rotational diffusion. We will remind in this section some of the basic aspects of rotational diffusion.

**Figure 1:**
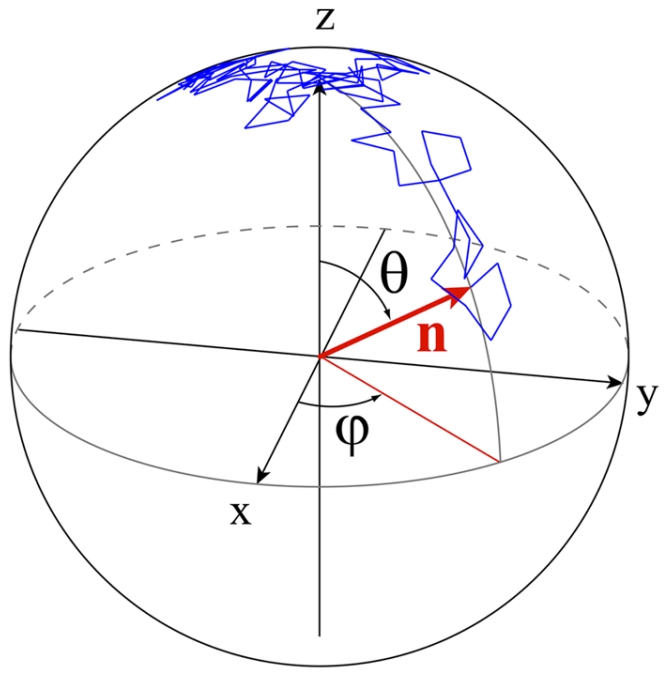
Random walk on a sphere. Trajectory (blue) of the extremity of a unit vector **n** (in red) describing a rotational Brownian motion. Initially the vector is aligned with the z direction (*θ = 0*). Each blue segment represents the angle variation 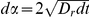 of this unit vector where *D_r_ = 0.16 rad^2^/s* (the rotational diffusion coefficient for a sphere of radius *1 µm* in a liquid of viscosity *η = 1 mPa.s* at ambient temperature T is 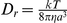 [17]). The time step is *dt = 30 ms* (total of 100 steps).

If *p(θ,ϕ,t)* is the probability density distribution for the particle to have an orientation of polar angle *θ* and azimuthal angle *ϕ* at a given time *t* (see fig. 1 for notations), the temporal evolution of this probability distribution is given by the rotational counterpart of the translational Fick's law [16]:
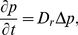
(1)where *D_r_* has units of rad^2^/s. It is the rotational diffusion coefficient of the particle [17].

Starting at t = 0, from a Dirac distribution *p(θ,ϕ,t = 0) = δ(θ)* (alignement of the particle with the polar axis Oz), the temporal evolution is given by an expansion in spherical harmonics [18]:
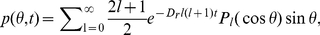
(2)where *P_l_* is the Legendre's polynomial of order *l*.

A simulation of this rotational diffusion process for a particle is shown in fig. 1. The extremity of a unit vector **n** indicating the orientation (major axis) of the particle describes a random walk on a sphere.

**Figure 2:**
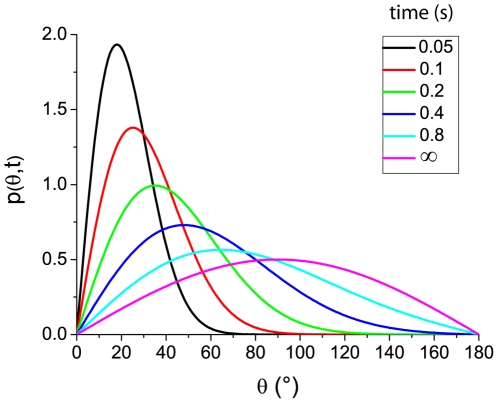
Distribution of reorientations during rotational diffusion. Evolution with time (from *t = 0.05 s to ∞*, cf. color code) of the reorientation distribution starting from the initial condition *p(θ,t = 0) = δ(θ)* (*i.e.* eq.2 for different values of *t*). The rotational diffusion is *D_r_ = 1 rad^2^/s*. For 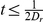, the particles keep in part the memory of their orientations whereas for 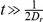, the orientation is almost completely randomized: 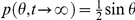 (pink curve).

The directional correlation function *<cosθ>(t) = <* ***n*** *(t)·* ***n*** *(0)>* deduced from this distribution is simply given by
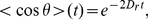
(3)and shows that at short times (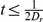), the particle keeps the memory of its orientation whereas at longer time (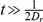) the orientation is completely randomized and leads to 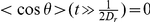 (Corresponding to a probability distribution 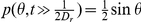 *i.e.* the only contribution in Eq.2 is *P_0_ = 1* (fig. 2, pink curve)).

### Distribution of reorientations during bacterial tumbles

**Figure 3:**
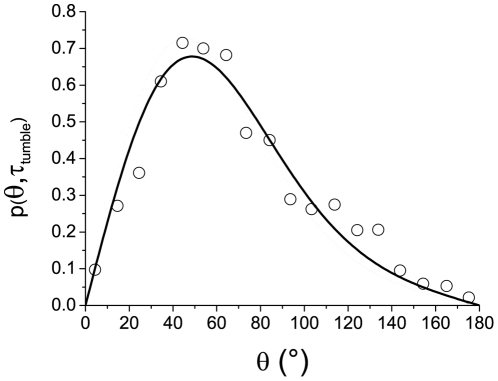
Distribution of reorientation during bacterial tumbles. Reorientation distribution (circles) during tumbles (obtained on about 1200 tumble events, from Berg and Brown [7]) and best fit (solid line) obtained from Eq.2 corresponding to *D_r_ = 3.5 rad^2^/s*. The tumble times are described by a discrete exponential distribution (mean value *<τ_tumble_> = 0.14 s*, time step *Δτ_tumble_ = 0.1 s*, same conditions as in [7]). The distribution of reorientation is well described by a rotational diffusion process.

We question here whether tumbles can be described by active rotational diffusion in the reorientation space (*θ*). The distribution *p(θ)* of the reorientation angles between successive runs has been studied in [7] and is reported in fig. 3 (circles). The curve exhibits a maximum close to *θ* = *63°* meaning that the orientation of the bacteria is not completely randomized during a tumble (*<θ> = 69°*). There is therefore a persistence in the orientation from one run to the next. The duration of these tumbles is exponentially distributed with a mean value of *<τ_tumble_> = 0.14 s* [6].

If the bacteria tumbles can be modeled by a process of active rotational diffusion, the result of averaging multiple reorientations of independent bacteria as was performed in [7] should also be described by equations (2) and (3). Therefore, assuming a unique coefficient of effective rotational diffusion for all bacteria tracked in these experiments, we can fit the experimental points by the function *p(θ,τ_tumble_)* from eq.2. To follow the analysis reported in [7], the tumble times *τ_tumble_* are described by a discrete exponential distribution mimicking the experimental procedure (mean value *<τ_tumble_> = 0.14 s, time step Δτ_tumble_ = 0.1 s*). The theoretical curve that best fits the experimental data is obtained for a rotational diffusion coefficient *D_r_ = 3.5±0.3 rad^2^/s* and the agreement is then very good (fig. 3), confirming *a posteriori* our initial assumption.

**Figure 4:**
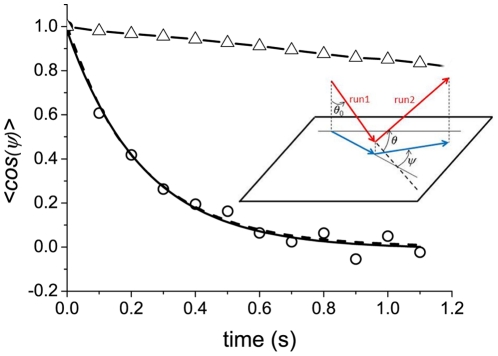
Temporal autocorrelation function *<cosψ>* versus tumble times. The projected directional correlation functions as a function of tumble durations are obtained from the analysis of the trajectories of swimming bacteria in motility buffer (black circles). These experimental points are fitted by a single decaying exponential (solid line) or the function given in eq.4 (dashed line). Both fits give the same values (within 10%) for the rotational diffusion coefficient *D_r_ = 2.1±0.3 rad^2^/s*. The calculated directional correlation function for beads of hydrodynamic radius *1 µm* is also shown (triangles) as a reference (see fig. 1 and [17]). **Inset: Notations used for the angle measured in 2D.** The red arrows represent two successive runs forming an angle *θ* in 3D. The corresponding projections in the observation plane (blue arrows) form a 2D angle *ψ* measured experimentally.

The observation of bacterial trajectories in the absence of gradient allows to measure the temporal evolution of the reorientations. Fluorescent bacteria suspended in motility buffer and enclosed in a small chamber were observed and tracked under a low magnification fluorescence stereomicroscope. Using a homemade software, the trajectories were decomposed in runs and tumbles and the changes in orientation between successive runs were measured as a function of the tumble duration [15]. Here, the reorientations were measured in the observation plane (see fig. 4 inset) and not in 3D. We denote *ψ* the projected reorientation angles of the bacteria in this plane.

The exact expression of the 2D projection (see fig. 4 inset for notations) of eq.3 is given by
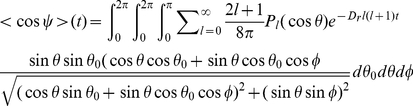
(4)Even if this expression can be used directly to estimate *D_r_*. The simplified expression 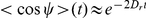 (corresponding to a second order approximation of eq.4) is accurate within 5–10% (fig. 4) and can be used as well to determine the rotational diffusion coefficient. We obtained a value of *D_r_ = 2.1±0.3 rad^2^/s* (fig. 4).

Since our experiments were performed in Motility Buffer (MB), a direct comparison with [7] is difficult (media and bacterial strains are different), but the diffusion coefficients are of the same order of magnitude and in any case, two orders of magnitude larger than passive diffusion coefficients of colloids of comparable size.

### Temporal evolution of the orientation during a tumble

The analysis of bacteria trajectories gives the tumble durations and the corresponding change in orientation (the angle between two successive runs) without details on the evolution of the orientation during a tumble. Moreover, since the tumble durations are exponentially distributed [19], the probability of long tumbles was very small. To track the change in orientation during a tumble, we have performed direct reorientation measurements on “tumbler” mutants deleted in the protein CheZ (strain CR33, ΔCheZ). These bacteria always maintain a high level of CheY-P (phosphorylated CheY protein) and thus always tumble [20], [21]. Furthermore, these mutants have basically no translational velocity and stay in the observation plane for a long time compared to the characteristic time of rotational diffusion 
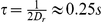
.

**Figure 5:**
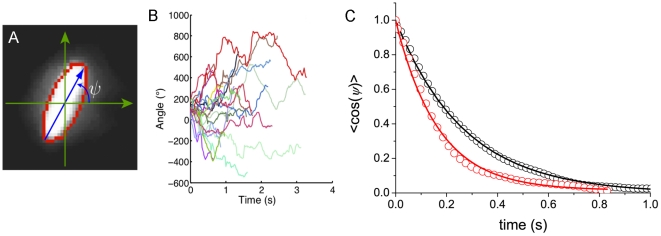
**A**) **Microscopic analysis of the bacterial tumbles.** The major axis of a fluorescent bacterium in projection in the observation plane of a microscope forms an angle *ψ* with a direction of reference (image width 5 µm).*ψ* was measured experimentally by fitting the projected shape by an ellipse. These experiments were performed on bacteria that always tumble (“tumbler” strain CR33) (see Movie S1). **B**) **Temporal evolution of the orientation.** Angle *ψ* (in the observation plane) of the major axis of the bacteria (for 20 different tumblers) as a function of time. **C**) **Temporal autocorrelation function versus time.** The projected directional correlation functions were obtained for “tumbler mutants” in LB medium (red circles) and in M9 medium (black circles). These experimental curves were fitted (solid lines) by a single exponential and give respectively for the only adjustable parameter *D_r_^LB^ = 2.4 rad^2^/s* and *D_r_^M9^ = 1.6 rad^2^/s*.

Here, we determine precisely the evolution of the orientation of an ellipse-shaped bacterium as a function of time (fig. 5A and B) by high frequency observations at high magnification.

Fig. 5C shows the directional correlation functions versus time *<cosψ>(τ) = <cos(ψ(t+τ)−ψ(t))>* of tumbler mutants in LB and M9 medium. The values obtained for the rotational diffusion coefficients in LB and M9 medium are respectively *D_r_^LB^ = 2.4 rad^2^/s* and *D_r_^M9^ = 1.6 rad^2^/s*. Again, even though the strains are different, these values are very close to the ones determined by analyzing the trajectories of the motile *E. coli* and confirm that the reorientation of the bacteria can be described by an active rotational diffusion process.

### Tumble modulations in a D-glucose gradient

**Figure 6:**
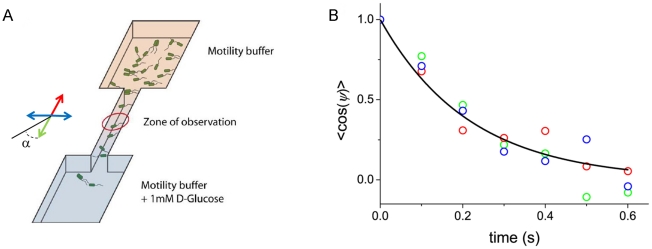
Modulation of reorientations in a fixed D-glucose gradient. A) **The experimental setup** consisted in two wide chambers connected by a long and narrow channel (6 mm×0.7 mm). One of the chambers was filled with motility buffer with 1 mM D-glucose and the other with a very dilute suspension of motile and fluorescent bacteria previously cultured at 30°C in LB to an OD of 0.5, and then diluted in motility buffer to an OD of 0.005. At these low concentrations, consumption of nutrients or oxygen is negligible. The trajectories were collected thirty minutes after inoculation, in the middle of the channel and analyzed following the procedure described in [15]. The color code stands for the four quadrants according to direction of the gradient. B) **Temporal autocorrelation function** ***<cosψ>*** between runs versus time in a D-glucose gradient. The color code (green, red blue) divides the plane in four main quadrants and defines the direction of the run preceding the reorientation (cf. fig. 6A). The exponential curve (black line) corresponds to the rotational diffusion coefficient obtained in the Glucose-free medium *D_r_ = 2.3±0.3 rad^2^/s* (fig. 4) and agrees well with the experimental points. The value of *D_r_* does not depend on the swimming direction relatively to the gradient.

We have described so far the behavior of bacteria in isotropic environments. In a gradient of nutrients (fig. 6A), one can observe a mean chemotactic drift velocity *V_drift_ = 5.1±0.5 µm/s* of the bacteria along the gradient. We [15], and others [14], have shown recently that chemotactic gradients affect both the runs and the tumbles: on top of the classically observed modulation of the runs durations with respect to the swimming direction of the bacteria, bacteria tended to reorient less, with smaller tumbles times, after a run in the favorable direction.

This amplitude of reorientation is given by the product of the tumble time by the diffusion coefficient. We have shown already that the tumble time was modulated according to the orientation of the bacterium [15], we now address the question whether this process is compatible with a single rotational diffusion coefficient. To that end, we plot the directional correlation function for the three directions of the run preceding the reorientation: up-the-gradient (green), down-the-gradient (red) and perpendicular to it (blue) (fig. 6A). We observe that these three correlation functions collapse on a single curve well fitted by the unique diffusion coefficient *D_r_ = 2.3±0.3 rad^2^/s* obtained in the absence of gradient (fig. 6B).

Since a unique diffusion coefficient describes well the tumbles in a gradient, the tumbles contribute to the chemotactic drift only by a differential adjustment of their duration (the diffusion time) according to their swimming direction. As a consequence, bacteria swimming up the gradient reorient less which improves the chemotactic drift [15].

### Tumble modulations in a bacterial wave

**Figure 7:**
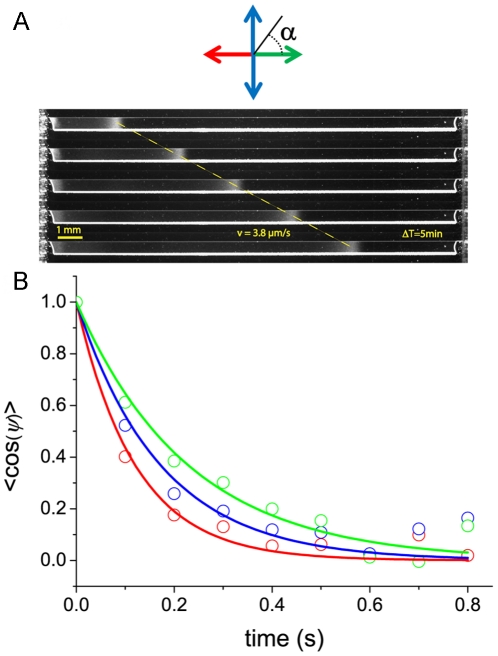
Modulation of reorientations in a propagating concentration wave. A) **Top view of a bacterial wave propagation.** Experiment performed in Minimal medium in a micro-channel (length *18 mm*, width *0.5 mm* and height *100 µm*). The channel was filled with a homogeneous suspension of bacteria (∼*5*×*10^8^cell/cm^3^*), closed at both ends and then centrifuged to accumulate bacteria at one end of the channel. After centrifugation, a bacterial wave forms and propagates at velocity *V_f_ = 3.8 µm/s*. The color code stands for the four quadrants according to the direction of wave propagation (See [15] for details). B) **Temporal autocorrelation function** ***<cosψ>*** between runs versus tumble times for bacteria within a wave. Again, the color code is used to subdivide the plane in four quadrants according to the direction of propagation (cf. fig. 7A). The corresponding rotational diffusion coefficients extracted from these data are *D_r_ = 2.3±0.3 rad^2^/s* (green), *D_r_ = 3.2±0.3 rad^2^/s* (blue) and *D_r_ = 4.4±0.3 rad^2^/s* (red).

Although a single diffusion coefficient can describe the tumbles in a shallow gradient, it is not clear if this is still the case in steeper ones. Such gradients are experienced in self-propagating concentration waves (fig. 7A, see also [22], [23], [24]). Individual trajectories of the bacteria within these waves have been recently studied in detail [15] and allow to access *<cosψ>(τ_tumble_)*. From the tumbles time distribution and reorientation, we get the corresponding rotational diffusion coefficient within the front (fig. 7B).

Here, the correlation functions were clearly different according to the swimming direction, each of them being described by an exponential decay of the reorientation as a function of time. When averaging out the values of the diffusion coefficients over the whole front, we found *D_r_ = 2.3±0.2 rad^2^/s* for tumbles occurring after a run in the direction of wave propagation (green), *D_r_ = 4.4±0.3 rad^2^/s* in the opposite direction (red), and *D_r_ = 3.2±0.2 rad^2^/s* in the perpendicular direction (blue) (fig. 7B). In this experiment, it is thus impossible to describe tumbles with a unique rotational diffusion coefficient and solely a modulation of tumble times. Here, the relative modulation of the rotational diffusion coefficient itself (*ΔD_r_/D_r_∼67%*) is more important than the variation of tumble durations (*Δτ_r_/τ_r_∼22%*) [15]. Nevertheless, both contributions act in synergy to modulate reorientations and improve the drift velocity of bacteria in the gradient.

## Discussion

The respective effects of run lengthenings and reorientations have been studied in details in [19]. The authors have shown that the active translational diffusion coefficient of a bacteria is affected by its mean reorientation angle (persistence) following,
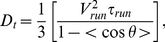
(4)where the mean reorientation angle between two successive runs is given by *<cosθ>∼0.33* [8].

Since the tumbles are well decribed by a rotational diffusion process, eq. (3) holds and leads to
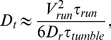
(5)showing that run and tumble modulations are equally weighted in the bias of the bacterial motion.

In the shallow glucose gradient experiment, the modulations of runs and tumbles are of the same order of magnitude (∼50%) and equally affect the translational diffusion coefficient. It is possible to quantify more precisely both contributions and to directly compare them with the measured value of *V_drift_*. The run lengthening effect alone would give a drift velocity 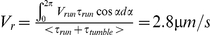 much smaller than the actual velocity *V_drift_ = 5.1 µm/s* (fig. 6). If we include the modulation of tumble durations or reorientations, we get 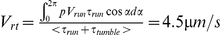 in much better agreement with the measured drift velocity of the bacteria. The contribution of the tumbles to the chemotactic drift amounts to more than 30% in this case.

We note that computer simulations have shown that a small (∼5°) modulation of the reorientation after a tumbles can dramatically affect the mean drift velocity, up to a factor of two [14]. It is therefore not surprising that the observed modulation of ca. 10° in the glucose gradient has a very significant contribution.

Although the same trend is observed for bacterial waves (*i.e.* opposing effects of the gradient on run lengthening and tumble shortening), a single diffusion coefficient cannot describes all the tumbles within the wave even though this description holds for subfamilies sorted upon their swimming direction. As proposed in [14], the number of flagella involved in the tumbles may vary with the direction of the preceding run, affecting directly the effective rotational diffusion coefficient.

In conclusion, we have shown that tumbles actively participate to chemotaxis of bacteria by a fine adjustement of their durations. In gradient-free situations or in shallow gradients the reorientation of the bacteria is well described by a rotational diffusion process and decays exponentially with tumble time. It is thus possible to introduce a rotationnal diffusion coefficient during tumbles of the order of a few rad^2^/s that characterizes this active reorientation process. Such a simplified description does not hold in steeper gradients where the number of flagella involved during the tumbles varies.

## Materials and Methods

### Bacterial strains

In this study, we have used the *E. coli* strain RP437 considered as wild-type for motility and chemotaxis experiments (kindly provided by J. S. Parkinson, University of Utah) and its derivative, a “tumbler” mutant (ΔCheZ) [21] (kindly provided by, C. Rao, University of Illinois).

### Plasmid

Both strains were transformed with a PZE1R-GFP plasmid (kindly provided by C. Beloin and J.-M. Ghigo, Institut Pasteur, France) [25], by preparing chemically competent cells using TSS buffer, and transforming them with the plasmid by heat shock [26].

### Bacterial culture

Different media were used in this study:

- M9 Minimal Salts, 5× supplemented with 1 g·L^−1^ Bacto™ Casamino Acids (both from Difco Laboratories, Sparks), 4 g·L^−1^ D-Glucose, and 1 mM MgSO_4_ [23].
- “Motility buffer” [23] was composed of 10 mM potassium phosphate buffer (pH 7.0) supplemented with 0.1 mM EDTA, 1 µM L-Methionine, and 10 mM sodium lactate. 1 mM D-Glucose was added to create the chemical gradient.
- LB medium (Sigma) was also occasionally used for bacterial culture when specified.

In all these experiments, cells were cultured at 30°C in 3 mL medium, shaken at 300 rpm up to reach an OD_600_ = 0.5 (∼5×10^8^ bact/cm^3^). Ampicilin (50 µg/mL) was added to maintain the plasmid when necessary.

### Experiments with tumbler mutants

The tumbler mutant cells (ΔCheZ) were grown either in LB medium or in enriched M9 medium up to an OD_600_ = 0.5. A droplet of this solution was entrapped between two coverslips separated by thin PDMS spacers (100 µm). Bacteria were observed at ambient temperature (22°C) under an inverted fluorescence microscope (Zeiss Axiovert 200) with a 100× objective and an intensified camera (Evolve, Photometrics) at 50 fps, approximately in the middle of the chamber. The resulting images were analyzed with the ImageJ plugin Analyze Particles to extract (best fit by an ellipse fig. 3B) the angle *ψ* of the major axis of the bacteria in the observation plane as a function of time (fig. 3C).

### Experiments with swimming bacteria

The microfluidic channels were placed under a variable zoom fluorescence stereomicroscope (MZ16FA, Leica—magnification range 0.8×–10×) equipped with a CCD camera (CoolSnapHQ, Roper Scientific). The whole set-up was maintained at 30°C, high humidity during the experiments to prevent evaporation through PDMS. Fluorescence mode was used to track the individual fluorescent bacteria. In our setup, the depth of field was ∼100 µm making possible to follow single bacteria over 20 s or more and measure the projections of their actual trajectories.

For the traveling wave experiments, low intensity transmission illumination (dark field mode) was superimposed to simultaneously follow the motion of the (mostly non-fluorescent bacteria mixed with fluorescent one at a ratio 500/1) wave [15]. Several one-minute long movies of the collective migration were acquired at 10 fps (WinView/32 software, Princeton Instruments).

## Supporting Information

Movie S1 **Movie of a “tumbler” mutant:** Motion of the “tumbler” strain (CR33) observed by fluorescence microscopy. This observation is performed at high magnification (×100, image width 5 µm) and high video rate (50 fps). The orientation of the bacterium in the focal plane is deduced by computing the major axis of the ellipse that best fits the image of the bacterium.
(AVI)
